# Public Emotional and Thematic Responses to Major Emergencies on Social Media, 2024-2025: Cross-Sectional Convergent Mixed Methods Study

**DOI:** 10.2196/84648

**Published:** 2026-01-20

**Authors:** Xingrong Guo, Yiqian Fan, Yiming Guo

**Affiliations:** 1 College of Foreign Languages Shanghai Maritime University Shanghai China; 2 School of Economics and Management Shanghai Maritime University Shanghai China

**Keywords:** emergencies, cross-culture, XLM-RoBERTa, BERTopic, emotion, topic, cross-lingual language model–robustly optimized BERT approach, Bidirectional Encoder Representations from Transformers Topic

## Abstract

**Background:**

During 2024-2025, global emergencies triggered intense online discourse, presenting a unique opportunity to examine how cultural factors shape emotional expression and knowledge dissemination. Understanding these dynamic mechanisms is crucial for enhancing the effectiveness of digital health communication and optimizing crisis response strategies.

**Objective:**

We analyzed how cultural and linguistic contexts influence emotional expression and thematic framing in social media comments during major emergencies in 2024-2025. We uncovered cross-cultural differences in collective emotions and narrative focuses, explaining how affective stance and discourse framing jointly shape the public construction of crisis meaning.

**Methods:**

We used a cross-sectional, convergent mixed methods design. Data were collected retrospectively from X (formerly Twitter; X Corp) and Weibo (Sina Weibo) between January 1 and December 31, 2024. Using purposive sampling, we selected 5-6 representative emergency events per month based on online visibility (capped at 600 comments/event). The dataset included 19,813 comments from X and 6536 comments from Weibo. Emotions were identified using a Cross-lingual Language Model-Robustly optimized Bidirectional Encoder Representations from Transformers approach, and thematic patterns were extracted with Bidirectional Encoder Representations from Transformers Topic. Integrated Gradients was used to interpret model outputs, while clustering and network analysis were applied to visualize cross-cultural patterns. Hofstede’s cultural dimensions theory helped interpret cultural influences on discourse. This mixed computational approach enabled a detailed comparison of emotional structures and thematic discourse across linguistic communities.

**Results:**

Significant cross-platform differences were observed in emotional distribution (*χ*²_8_=8025.60; *P*<.001). Compared to X users, Weibo users, representing a collectivist culture, expressed concentrated negative emotions (20.37%; odds ratio [OR] 15.76, 95% CI 13.90-17.85), surprise (19.70%; OR 2.53, 95% CI 2.32-2.73), and fear (16.68%; OR 1.72, 95% CI 1.58-1.86), reflecting group-oriented anxiety and emotional contagion. In contrast, X (formerly Twitter) users in individualist contexts displayed dispersed sarcasm (43.49%; OR 55.19, 95% CI 43.95-69.21) and worry (15.30%; OR 55.27, 95% CI 34.74-87.88), indicating personalized and critical emotional styles. Topic modeling revealed dense clusters around “safety,” “pray,” and “resettlement” on Weibo, whereas X (formerly Twitter) comments emphasized decentralized themes of critique and responsibility. Semantic network analysis revealed a cohesive fear-prayer-rescue chain on Weibo and fragmented, debate-oriented interactions on X (formerly Twitter).

**Conclusions:**

Emergency discourse is not neutral but is systematically structured by cultural values that shape emotions and themes. Integrating multilingual computational and qualitative methods, we offer a replicable framework using large-scale data, moving crisis and infodemiological research beyond single-platform or survey-based approaches. Our findings advance theory-informed understanding of how cultural meaning systems translate into observable digital discourse under conditions of risk and uncertainty. They also offer practical implications for governments, public health agencies, international organizations, and digital platforms by informing culturally adaptive, platform-specific risk communication, community moderation, and crisis engagement strategies that can strengthen public trust, improve compliance with protective behaviors, and mitigate infodemic-related harms.

## Introduction

### Background

This study investigates the emotional and thematic patterns exhibited in social media comments during key global emergencies spanning 2024-2025, focusing on how cultural factors mold emotional expression and information dissemination. Understanding these dynamic mechanisms is essential for improving digital health communication and crisis response strategies. Global natural disasters, public health crises, and human-made accidents have intensified in recent years, placing unprecedented demands on emergency communication and mental health support. Social media platforms now serve as real-time spaces for emotional expression and public discourse during such events [1]. For example, on May 1, 2024, a highway in Meizhou, Guangdong, collapsed after prolonged heavy rain and unstable geological conditions. The disaster caused 52 deaths and injured 30 people. Another tragedy occurred on December 29, 2024, when a plane crashed in South Korea. Only 2 passengers survived, while 179 passengers lost their lives. Both incidents generated millions of online reactions within hours.

Social media has increasingly been acknowledged as a vital tool in emergency management. It allows authorities to monitor public sentiment in real time, evaluate opinion-related risks, and enhance the quality of risk communication [2-4]. Surveys show that 75% of people use or plan to use social media during emergencies, and 77% believe it delivers information faster than traditional channels [5]. Weibo (Sina Weibo) and X (formerly Twitter; X Corp) spread emergency updates more quickly than conventional media and provide spaces for emotional exchange and debate [5].

Limited understanding exists of how different cultural groups emotionally respond to the same emergency through online comments [6]. Although many studies have explored the role of tweets or posts during emergencies [7,8], such as information dissemination, public emotion analysis of posts, and crisis communication strategies. User comments are often overlooked because they are more spontaneous and interaction-driven [9]. While a substantial portion of research has focused on single-language or single-region datasets, some cross-lingual studies exist [10,11]. However, these studies often do not fully explore cross-cultural differences in public responses, particularly in the context of emergencies across multiple social media platforms. Therefore, there remains a need for research that systematically examines cross-cultural emotional and thematic patterns in multilingual online discourse. To bridge this gap, the study draws on comments posted during major emergencies in 2024-2025 on Weibo and X (formerly Twitter), comparing how cultures differ in emotional expression and discourse patterns. Multilingual emotion recognition is performed with the Cross-lingual Language Model-Robustly optimized Bidirectional Encoder Representations from Transformers (BERT) approach (XLM-RoBERTa), while BERTopic modeling is used to cluster topics and trace emotions such as fear, anger, and sarcasm across cultures. By revealing how emotions are shaped in online discourse, the research explains the psychological factors behind users’ emergency responses and offers practical guidance for emergency communication that respects cultural differences.

The paper is structured as follows. Section 1 details data collection and the methods for preprocessing, emotion labeling, and topic modeling. Section 2 reports results on emotion patterns, topic clusters, and cross-cultural comparisons. Section 3 interprets these findings through cultural theories and discusses practical implications, concluding with limitations and directions for future work.

### Literature Review

#### Social Media as a Digital Health Communication Tool in Emergencies

Digital health, or the use of digital technologies for health, has become an important field that applies routine and new forms of information and communications technology to meet health needs [12]. Social media platforms such as Weibo and X (formerly Twitter) enable fast and interactive communication. People can share personal experiences, questions, and feelings in real time, adding to official announcements. This immediate feedback builds a shared understanding of a situation. It also helps officials respond to misinformation and ease public anxiety, making social media a key setting for digital health efforts [13-15].

Existing research has confirmed that during crises such as the COVID-19 pandemic, digital health communication can lower uncertainty and encourage protective behaviors [16]. Other studies show that effective online risk communication can reduce fear, prevent rumors, and improve a community’s ability to handle crises [17]. However, most of this research focuses on original posts or organizational messages. Less attention has been paid to the active and multilingual comment sections. In reality, comment sections often contain spontaneous, emotional conversations and reveal how people from different cultural backgrounds interpret health information. This content is crucial for understanding how people process risk information and decide how to respond. Despite the global reach of social media, there is still little cross-lingual and cross-cultural analysis of digital health communication.

#### The Unique Value of Comment Discourse in Emergency Communication

With the growth of social media, Weibo and X (formerly Twitter) have become major spaces for public emotion and opinion during crises. Unlike original posts that mainly broadcast information, comment threads allow real-time dialogue and shared narratives [18,19]. This bottom-up interaction captures immediate, authentic public sentiment [20] and reveals insights often missing from the posts themselves [19].

Comments play 2 key roles in society. They show natural emotional reactions and shape public agendas, influencing social norms and even public policy. Since discourse reflects cultural norms, emotional tones, and interaction styles vary across contexts [21]. For example, collectivist cultures stress emotional connection and group harmony, while individualist cultures focus more on self-expression [22]. Thus, comment styles on Weibo and X (formerly Twitter) during emergencies can differ, highlighting the need for cross-cultural comparison [23].

In social media research, comment sections have been less studied. One key reason is that these texts are often unstructured, fragmented, and full of everyday wording [24]. Nonstandard grammar, mixed languages, local slang, and subtle emotions further challenge traditional text analysis models in processing them [25,26]. These features make traditional models less effective, so studies on emergency communication have often left comments aside [27].

#### Emotional Characteristics and Multiemotion Analysis in Comments

Emotional expression is shaped by language and culture, as Dewaele and Pavlenko [28] emphasized in their cross-linguistic perspective on emotions, showing that different languages provide distinct repertoires for conveying feelings. In traditional sentiment analysis, Gul et al [2] note that it typically categorizes emotions into 3 broad types: positive, neutral, and negative. However, Kant et al [29] argue that this approach fails to capture the complexity of specific feelings such as fear, anger, worry, shock, and sarcasm, leading to an oversimplified understanding. Recent studies have shown that classifying emotions into 2 or 3 types is insufficient for crisis communication [4,30]. Regarding this issue, multiemotion labeling systems with categories such as fear, anger, and sarcasm offer a more accurate picture of public reactions and their potential policy implications [11,31].

Cross-lingual and cross-cultural sentiment analysis is still relatively understudied. Although social media is global, most research still relies on monolingual datasets and ignores culturally mixed communication [32]. Key linguistic characteristics, including the use of metaphors, styles of emotional expression, and cultural norms, differ greatly across languages [26]. These differences present challenges to current analytical models. Multilingual deep learning models such as XLM-RoBERTa can capture cross-linguistic semantic meanings [33,34]. Transformer-based topic modeling tools, such as BERTopic, demonstrate stronger performance in extracting coherent discussion themes from unstructured comment data infused with emotional content [35,36].

#### Existing Approaches in Social Media Emergency Research

Emergency communication research on social media mainly uses 3 methods: network analysis, content analysis, and sentiment analysis.

Network analysis studies how information spreads structurally during emergencies. For example, Zhang et al [37] showed that network structures affect how information spreads over time. Han et al [38] proposed a convolutional neural network with an extreme learning machine model-based algorithm to analyze the emotional influence of Weibo users. It measures how emotions spread among users. Singh and Singh [39] used text and graph multiview learning for tweet sentiment analysis, revealing structural and semantic connections. However, these kinds of studies place less emphasis on the content and emotions in messages.

Content analysis focuses on message features and communication strategies. Kada et al [40] analyzed government social media posts during COVID-19, and Chen and Ping [41] used the Wuli-Shili-Renli method for natural disasters. Nevertheless, content analysis has limitations in automating and scaling up when dealing with large amounts of user comments.

Sentiment analysis is widely used to gauge public emotions. Ou et al [30] explored the evolution of public sentiments, filling the gap in multiemotion classification studies. Studies [2,29] have found that negative feelings often dominate, while Halse et al [4] underscored their role in detecting trust. However, most sentiment studies only assess basic polarity (positive, negative, and neutral). Multiemotion classification remains scarce, especially in cross-lingual settings [32,42].

Recently, researchers have combined topic modeling methods with sentiment analysis to better capture themes and emotional tones. Babalola et al [43] reported that BERTopic is more effective than traditional models. Its usefulness in health-related social media studies was also confirmed by Khodeir and Elghannam [36] and Ma et al [44].

#### Hofstede’s Cultural Dimensions Framework

To analyze the discourse of cross-cultural comments, Hofstede’s [45] cultural dimensions theory is used in this study. It is a framework that not only compares the patterns of national cultures but also explains how culture influences comment style. Hofstede identifies 6 dimensions: power distance, individualism and collectivism, masculinity and femininity, uncertainty avoidance, long- and short-term orientation, and indulgence and restraint. These dimensions link cultural values to emotional and discursive behaviors. By applying Hofstede’s framework to the comparative analysis, this study aims to interpret emotional and thematic differences within broader cultural contexts.

Hofstede’s framework is applied in many areas beyond theory. It appears in studies on technology adoption [46], educational behavior [47], and online communication. Research [48] shows cultural dimensions such as individualism and power distance strongly affect behavioral intention and emotional expression across nations.

#### Research Gap and Study Contribution

Although research on emergency communication through social media has grown, 3 key gaps remain. First, comment sections have not been fully used. Their unstructured format and the complexity of analysis limit their practical application [24,25]. Second, most studies rely on monolingual datasets and seldom examine cross-cultural comment data [32,49]. Third, research on multiemotion sentiment analysis in cross-cultural emergencies is still scarce [11,30].

To fill these gaps, this study proposes an integrated framework. The framework combines BERTopic and XLM-RoBERTa to perform cross-cultural, multiemotion analysis of comments from Weibo and X (formerly Twitter) during emergencies. Its purpose is to expand research methods in emergency communication and to broaden the scope of discourse analysis in digital public spaces. It emphasizes both multilingual coverage and emotional sensitivity.

### Research Questions

This study examines user comments on Weibo and X (formerly Twitter) from multiple countries during 2024-2025 emergency events, focusing on emotional structures and thematic discourse. The analysis seeks to identify key topics and emotional patterns within social media comments and to explore how cultural factors shape styles of expression. To address these aims, XLM-RoBERTa is applied for sentiment analysis and BERTopic for topic modeling, providing data that enable detailed cross-cultural comparison.

This study seeks to explore the following research questions:

- RQ1: How do social media comments on emergency events express collective emotions and construct shared meanings?
- RQ2: In what ways do emotional expressions and narrative focuses differ across cultural and linguistic communities?
- RQ3: How are emotional valence and narrative focus interrelated in shaping the cross-cultural representation of emergencies?

Guided by these questions, the study proposes that the distribution and valence of emotional expressions differ significantly across cultural groups, reflecting distinct affective orientations and underlying value systems. Linguistic communities are also expected to demonstrate culturally specific narrative focuses when interpreting emergency events, revealing divergent framing patterns in emotional discourse. Moreover, emotional valence and narrative focus are assumed to be interrelated across cultures, suggesting that affective stance and discourse framing jointly contribute to the construction of crisis meaning.

## Methods

### Mixed Methods Design Overview

This study used a convergent mixed methods design, integrating quantitative computational analyses with qualitative content and discourse analysis to examine cross-cultural emotional and thematic patterns in social media responses to emergencies. The quantitative phase included multilingual emotion classification, frequency statistics, topic modelling, semantic co-occurrence analysis, and statistical testing. The qualitative phase involved manual coding by trained human coders, interpretive examination of representative comments, and theory-driven discourse analysis based on Hofstede’s cultural dimensions. Both strands were conducted in parallel, and findings were integrated during interpretation to triangulate results and enhance validity. In adherence with best practices for observational studies, this paper was drafted using the JARS (Journal Article Reporting Standards) guideline [50] and was edited according to the JARS reporting checklist [51], which is included in Multimedia Appendix 1 Checklist 1.

### Quantitative Component

#### Study Design and Data Collection

To explore public responses to emergencies, this study used a cross-sectional observational design and systematically collected social media comments from Weibo and X (formerly Twitter) between January 1 and December 31, 2024. At the end of each month, events from the previous month were systematically reviewed within a 1-week window. Continuous or nonbreaking events were excluded to ensure that only discrete emergency events were included in the dataset. Furthermore, the total number of comments across all major relevant hashtags for that event on Weibo and X (formerly Twitter) did not** **exceed 600 during the selected evaluation period. Given the potentially large data volume and uneven discussion intensity across events, a purposive sampling method was used. Each month, 5-6 representative events were selected based on topic relevance, comment volume, and overall online visibility, measured by comment counts, repost numbers, and trending hashtag rankings. Data were collected retrospectively after each event. Within the archived datasets, iterative sampling was conducted in successive batches until no new emotional categories or thematic patterns emerged, indicating the achievement of analytical saturation. This approach ensured that the dataset captured events that generated substantial public interaction and emotional expression on both platforms. For example, regarding the January 2024 Japan earthquake, specific trending hashtags were identified, including “# 日本地震 (Earthquake in Japan)” on Weibo and “#JapanEarthquake2024” on X (formerly Twitter). Posts related to these events were then systematically collected, with data acquisition carried out via custom Python scripts (Python Software Foundation), strictly adhering to the platforms’ developer agreement and used solely for noncommercial, academic research purposes.

In total, 26,349 valid comments were gathered through this process, with 19,813 comments from X (formerly Twitter) and 6536 from Weibo. The combination of random sampling, multistage cleaning, and independent coding ensured that the final dataset remained representative, reproducible, and reliable for analysis.

#### Measures, Predictors, and Confounders

This study focuses on emotional patterns and thematic content reflected in cross-cultural public comments. The XLM-RoBERTa model was used, achieving an accuracy of 78.37%, outperforming traditional models, including the support vector machine (64.2% accuracy) and XGBoost (62% accuracy) [52]. To better capture emotions, the quantitative results went beyond basic labels (“negative,” “positive,” and “neutral”) and included additional categories such as “worried,” “fear,” “angry,” “sarcasm,” “shock,” and “sad.” They also captured thematic structures and semantic network forms identified by computational models. In comparative analysis, platform type (Weibo and X, formerly Twitter) and event category (natural disasters and public crises) were regarded as key influencing factors. The study aimed to examine their differentiated effects on online emotional expression and discourse construction through analyzing these factors. In addition, several background variables were considered, covering event scale, total number of comments, posting time, and geographic origin. Quantitative processing of these variables was designed to ensure the statistical robustness of the main findings.

#### Data Processing and Sampling Procedures

**Figure 1:**
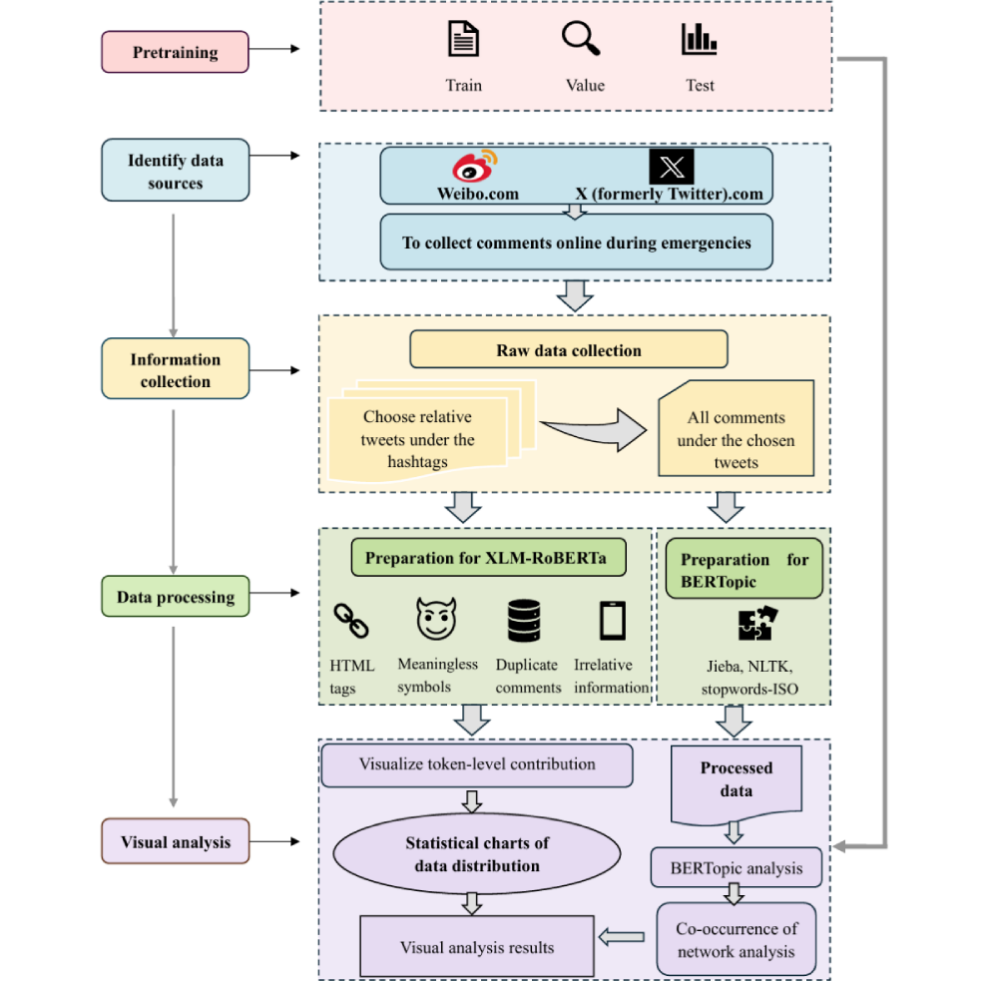
Data analysis procedure. The data analysis included 5 steps: pretraining data, collecting comments via Python, processing comments, model preparation, and building models for visual analysis. BERTopic: Bidirectional Encoder Representations from Transformers Topic; HTML: HyperText Markup Language; ISO: International Organization for Standardization; NLTK: Natural Language Toolkit; XLM-ROBERTa: Cross-lingual Language Model–Robustly Optimized BERT Approach.

As shown in Figure 1, the analysis proceeded in 5 major stages: pretraining, information collection, data processing, model preparation, and visual analysis. A multistage cleaning process was adopted, combining automated filtering and manual checks to eliminate spam, blank comments, advertisements, and duplicates. The collected data mainly included user IDs, posting times, usernames, comment content, repost counts, comment counts, such as counts and geographic locations. Due to the exclusion of invalid and incomplete entries during preprocessing (such as blank text content or missing timestamps), the final analytical dataset (N=26,349) consisted of complete cases, making multiple imputation unnecessary. The data were cleaned and stop words removed using different natural language processing libraries depending on language (Jieba for Chinese, Natural Language Toolkit for European languages, and stopwords-ISO [International Organization for Standardization] for others). The multistage cleaning process also involved eliminating irrelevant information, HTML tags, meaningless symbols, and duplicate comments. All personally identifiable information was destroyed immediately after collection, and only valid data was retained. For events exceeding 600 comments, Python random sampling was used to cap the dataset per event to maintain representativeness and reduce class imbalance.

#### Computational Modeling for Sentiment Classification and Topic Clustering

Following the multilingual fine-tuning approach outlined by Rasool et al [53], the study used the XLM-RoBERTa-base model using a learning rate of 2×10^–5^, batch size of 16, and 15 training epochs. The optimizer was AdamW with fused precision optimization adamw_torch_fused and a linear learning rate scheduler. The maximum sequence length was set to 128 tokens. Mixed-precision training (FP16) was enabled to optimize memory usage and training efficiency. To address class imbalance across emotion labels, a weighted binary cross-entropy loss function was used, with class weights inversely proportional to their frequency in the training corpus. Continuous emotion probability scores were binned into discrete emotional labels using predefined thresholds (neutral=0.12, surprise=0.10, positive=0.25, negative=0.30, sarcasm=0.25, fear=0.20, sad=0.25, worried=0.25, and anger=0.28). A total of 11,933 sentences were used for pretraining [54] to help the model better handle specific emotions, where meanings differ from literal words [55].

To mitigate potential semantic misalignment for low-resource languages (eg, Hindi and Indonesian), the research adopted a back-translation data augmentation strategy using the Many-to-Many 100 multilingual translation model. It has been shown to improve model quality in low-resource languages [56]. Each comment was translated from its source language to English and then back to the original language, enriching contextual diversity and improving cross-lingual embedding alignment before fine-tuning.

**Figure 2:**
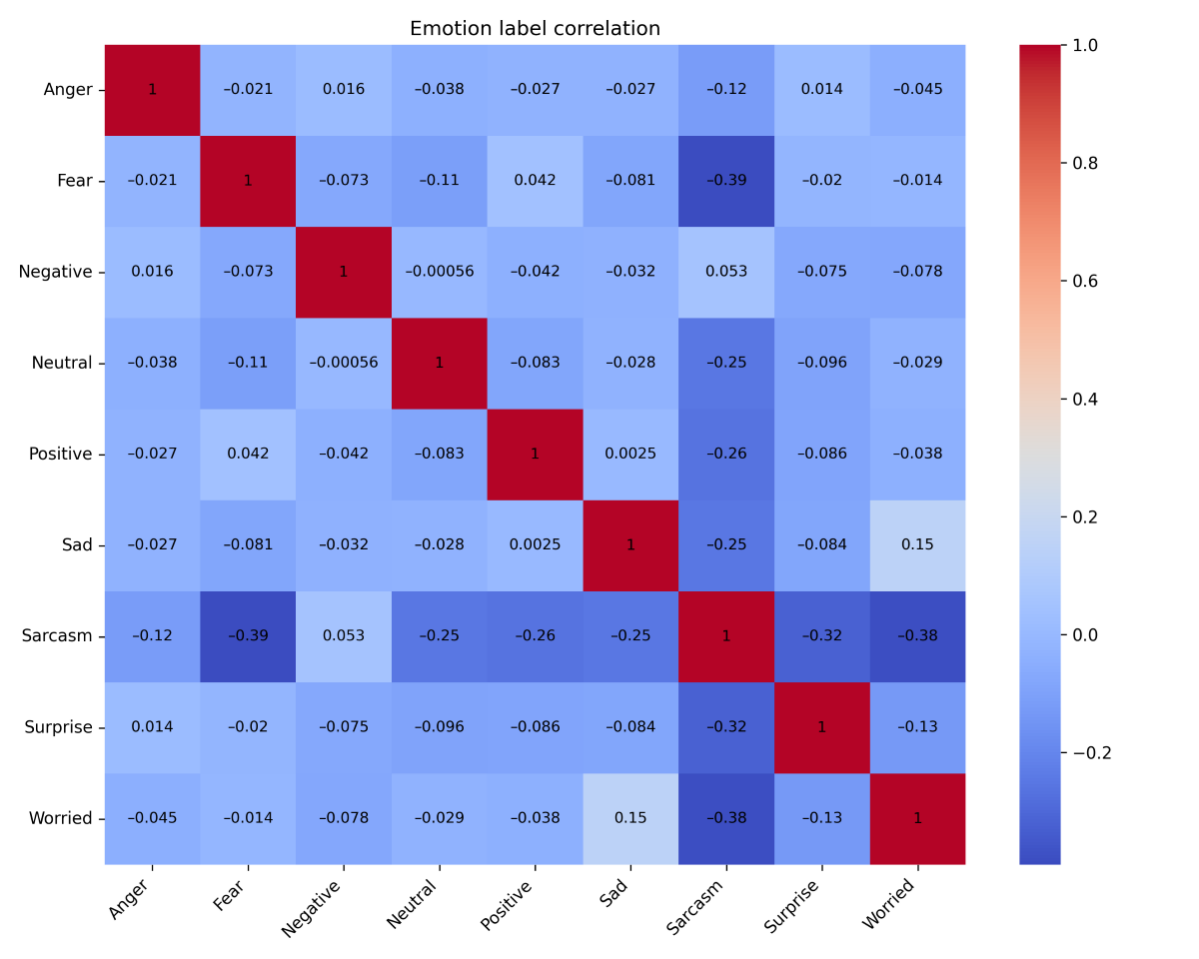
Correlation matrix of the 9 identified emotion labels. The heatmap displays the Pearson correlation coefficients (r) between all emotion pairs, demonstrating generally low linear correlation across the labels.

XLM-RoBERTa was applied to real data only after it showed adequate performance. As shown in Figure 2, the correlation checks between labels (ranging from –0.39 to 0.15) confirmed low overlap, indicating that the model has sufficient ability to process text. In addition, a manual verification step was conducted to ensure classification accuracy and validate the model’s reliability. Two trained coders independently analyzed a random sample of 300 comments (150 from Weibo and 150 from X) to check the alignment between automated labels and human interpretation. After 2 coding rounds, the inter-rater reliability (Cohen κ) reached 0.88, confirming the model’s high precision in capturing emotional nuances across both platforms. Nevertheless, because the model relies primarily on linguistic and semantic patterns without incorporating broader contextual cues, it may not fully capture context-dependent expressions of sarcasm or other nuanced emotional tones. These methodological constraints were taken into account when interpreting the results.

**Figure 3:**
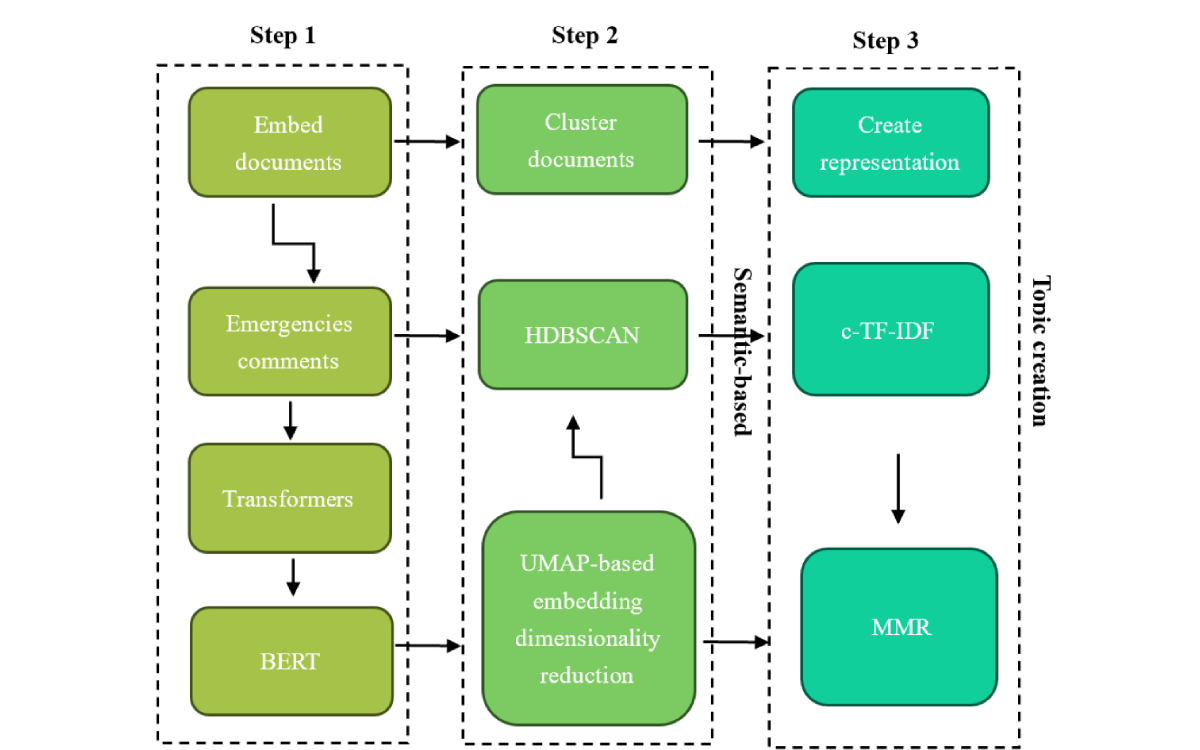
Bidirectional Encoder Representations from Transformers Topic (BERTopic) modeling workflow for extracting topics from social media comments. The process consists of three main steps: (1) document embedding using Bidirectional Encoder Representations from Transformers (BERT) and Transformers, (2) document clustering using Uniform Manifold Approximation and Projection (UMAP) for dimensionality reduction and Hierarchical Density-Based Spatial Clustering of Applications with Noise (HDBSCAN), and (3) topic creation via class-based term frequency-inverse document frequency (c-TF-IDF) and Maximal Marginal Relevance to generate topic representations and keywords.

For topic clustering, the study adopted the BERTopic framework, which outperforms traditional models such as latent Dirichlet allocation and nonnegative matrix factorization when handling short texts [57]. Grootendorst [58] upgraded BERTopic, and this study adopted his improved version. As shown in Figure 3, BERTopic combines BERT embeddings, UMAP for dimensionality reduction, and HDBSCAN clustering to extract key topics from comments, followed by class-based term frequency-inverse document frequency (c-TF-IDF) to generate keywords for each topic.

#### Statistical Analysis

Descriptive statistics were used to summarize distributions of comments and emotion categories. Proportions were reported with their 95% CIs, which served to quantify the precision of the parameter estimates for the analyzed dataset. Chi-square tests were conducted to assess cross-platform differences. Correlation matrices were generated to evaluate overlap among emotion labels. Statistical charts were created to visualize data distributions.

#### Model Interpretability Analysis

To address the inherent black-box nature of the fine-tuned XLM-RoBERTa model, the study used the Integrated Gradients (IG) attribution method [59]. IG attributes the model’s prediction to input features by calculating the path integral of the gradient from a baseline (zero embedding) to the actual input. It satisfies the axioms of sensitivity (changes to an essential input feature must lead to a change in attribution) and implementation invariance (attributions must be independent of the specific model implementation) [60].

The IG attribution score for an input feature is computed as follows [61]:



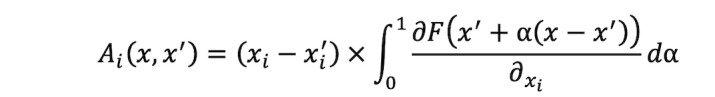



Where F(x) is the model’s prediction function (the target logit), is the input embedding, and χ′ is the baseline embedding.

For the XLM-RoBERTa implementation, the attribution target was set to the logit output corresponding to the predicted emotion category. The baseline input “χ′” was defined as the zero embedding vector. This vector represents the absence of textual information and is a standard practice in natural language processing interpretation. The path integral required by IG was numerically approximated using the Riemann sum with 50 steps. A balance between computational efficiency and convergence accuracy was achieved with this setting. Since XLM-RoBERTa uses high-dimensional token embeddings, the raw IG output is a vector for each subword token. The final scalar token-level attribution score was derived by averaging the contribution across all dimensions of the embedding vector. This process yields a single value, which clearly indicates whether a token supports (positive score) or suppresses (negative score) the classification decision.

### Qualitative Component

#### Data Sources and Sampling

The qualitative component was based on the same corpus of publicly available social media comments analyzed in the quantitative phase. Rather than generating new qualitative data or recruiting participants, this study examined naturally occurring textual data posted on Weibo and X (formerly Twitter) in response to emergency events during 2024-2025. These comments represent unsolicited public expressions produced in real-world digital environments and were therefore treated as textual data sources rather than participant-generated responses.

Qualitative analysis focused on interpreting how emotions and meanings were discursively constructed within the broader patterns identified by computational analysis. Representative excerpts were examined to contextualize emotion categories, topic structures, and semantic relationships observed at the aggregate level. This approach allowed qualitative interpretation to be embedded within the full dataset, supporting explanation and triangulation without introducing a separate qualitative sample.

#### Manual Coding Procedures

To ensure the methodological integrity of the qualitative discourse analysis, the study conducted a separate theoretical verification. The verification was meant to systematically link emotional themes to Hofstede’s cultural dimensions through the emotion-culture mapping framework. Two coders independently analyzed representative comments for theory-driven mapping. The intercoder reliability for this thematic categorization reached a Cohen κ of 0.82. Coding focused on the dominant semantic meaning and pragmatic context, such as sarcasm. This approach helped capture cultural nuance. Any discrepancies were resolved through discussion, and full consensus was reached to provide an empirically supported foundation for cross-cultural interpretations.

#### Discourse and Pragmatic Analysis

Linguistic differences help reveal cultural disparities. To explain how emotions and comment styles differ across cultures, Hofstede’s cultural dimensions theory [45] is applied in this study. For instance, expressions such as “congratulations” in an emergency context were interpreted not as positive but as having a “sarcastic” connotation. Representative comments were analyzed at the semantic level to explain how emotions and comment styles differ between the collectivist context (Weibo) and the individualist context (X, formerly Twitter).

### Integration of Quantitative and Qualitative Strands

The study combined findings by linking emotional tone (quantitative) with narrative focus (topic modeling). This mixed methods approach showed how affective orientation and discourse framing together shaped cross-cultural meaning construction. Semantic co-occurrence network analysis was combined with statistical distributions. The research emphasized the interaction between emotion and narrative structure and demonstrated that distinct emotional tendencies matched particular thematic patterns in multilingual emergency communication.

#### Reflexivity and Research Stance

The study acknowledges that, while the model relies on linguistic patterns, it may not fully capture all context-dependent expressions. Therefore, methodological constraints were taken into account when interpreting the results. The interpretation of “cultural disparities” is framed through the specific lens of Hofstede’s theory, acknowledging the distinction between the Chinese cultural context and the Western-dominated context of X (formerly Twitter).

### Ethical Considerations

This study drew on publicly accessible comments about 2024-2025 emergency events from X (formerly Twitter) and Weibo. All data were collected with Python scripts, following the platforms’ terms of service. The study was deemed exempt from formal human research ethics approval by institutional guidelines, as it involved no direct interaction with individuals and used only publicly available data. Informed consent was not required since the data were public, and platform policies already notify users of potential academic use. To ensure privacy, user identifiers (such as usernames and IP addresses) were collected but immediately discarded, and all textual content was thoroughly anonymized by removing indirect identifiers before analysis. In addition, no images, figures, examples, or supplementary materials included in this paper contain information that could lead to the identification of individual users. All illustrative excerpts and visualizations are fully anonymized and presented in aggregate form. Therefore, no identifiable personal data are disclosed, and additional individual consent was not required. The research strictly followed the ethical principles of the Declaration of Helsinki and complied with major data protection regulations, including the General Data Protection Regulation and the Chinese Cybersecurity Law.

## Results

### Interpretability Analysis

**Figure 4:**
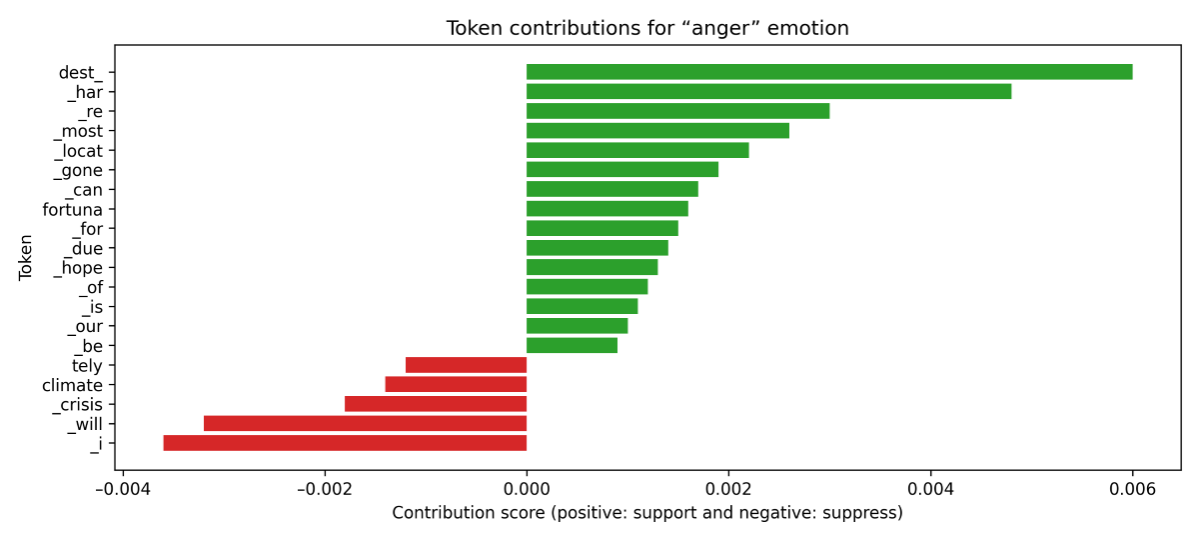
Integrated Gradients (IG) token contribution scores for the “Anger” emotion, validating the Cross-lingual Language Model–Robustly optimized BERT approach (XLM-RoBERTa) model. The bar chart displays tokens that positively (green) or negatively (red) contribute to the model’s prediction of “anger”.

**Figure 5:**
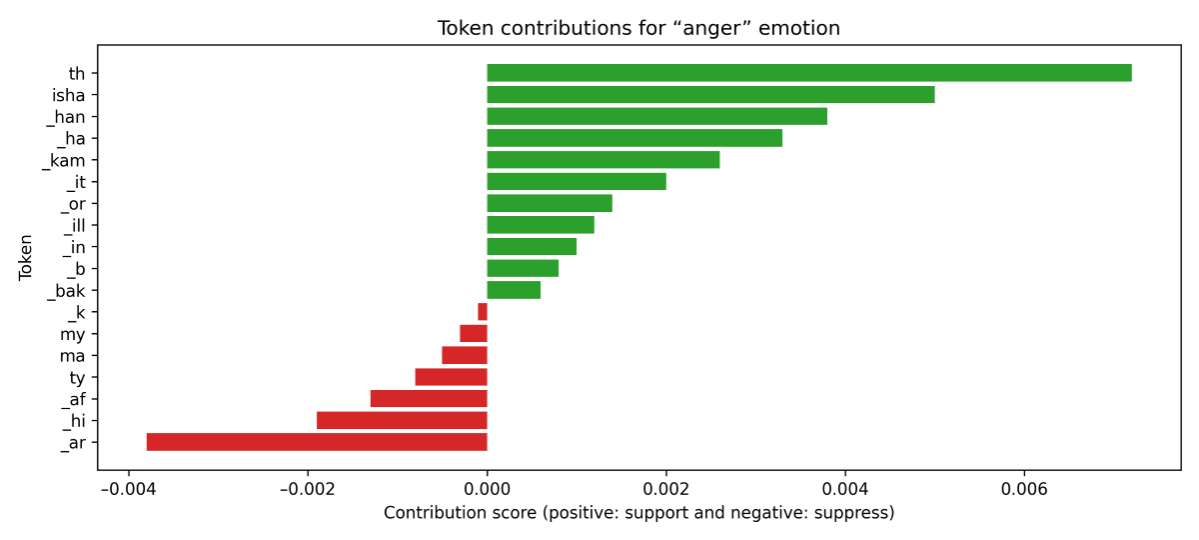
Integrated Gradients (IG) token contribution scores for the “Worried” emotion, validating the Cross-lingual Language Model–Robustly optimized BERT approach (XLM-RoBERTa) model. The bar chart shows the positive (green) and negative (red) contribution scores of key tokens.

A comprehensive interpretability analysis was conducted for the 9 emotion labels. The analysis consistently demonstrated that the XLM-RoBERTa model’s predictions were highly dependent on tokens with explicit emotional valence, matching established principles of affective linguistics. As examples, local attribution results for the “worried” and “anger” emotion classifications are shown in Figures 4 and 5. For predicting “worried,” the model relies heavily on specific entities or situations that may pose harm. Tokens with the highest positive contributions, such as “dest” (destination), “_har” (hardship or harm), and “locat,” point to entities or difficulties that clearly strengthen the model’s judgment of worry.

For predicting “anger,” decisions by the model depend strongly on tokens representing highly critical or aggressive language. The tokens with the highest attribution scores, including _h, isha, and _han, correspond to strongly negative words and serve as key evidence for XLM-RoBERTa to identify anger.

In conclusion, the IG interpretability analysis provides direct evidence validating that XLM-RoBERTa’s decision-making mechanism is trustworthy and intuitive. This subword-level analysis reveals that the model effectively distinguishes between relying on specific, contextual tokens and relying on strong, affective tokens, significantly enhancing transparency and trust in the model’s operations.

### Emotional Distribution and Cultural Difference

Understanding how people feel about emergencies helps us understand how the public perceives risks and their attitudes toward them. This helps policymakers make timely decisions that consider culture. These decisions improve social stability. To test whether there is a significant difference, *P*<.001, in the distribution of emotional labels between 2 platforms, a chi-square test of independence was conducted. As shown in Table 1, the results show that there is a highly significant association between the platforms and the distribution of emotional labels. This indicates that there are obvious statistical differences in the proportions of different emotions expressed by users on Weibo and X (formerly Twitter).

**Table 1:** Statistical differences in the proportion of emotional labels between Weibo and X (formerly Twitter). The results (χ²8=8025.60; *P*<.001) indicate a highly significant association between the platform used and the type of emotional labels expressed by users. All expected cell counts exceeded the minimum requirement for chi-square analysis (181.76).

Test statistic	Value (*df*)	Asymptotic significance (2-sided)
Pearson chi-square	8025.598 (8)	<.001
Likelihood ratio	8551.340 (8)	<.001
Linear-by-linear association	3008.296 (1)	<.001
N (valid cases)	29040	—

**Figure 6:**
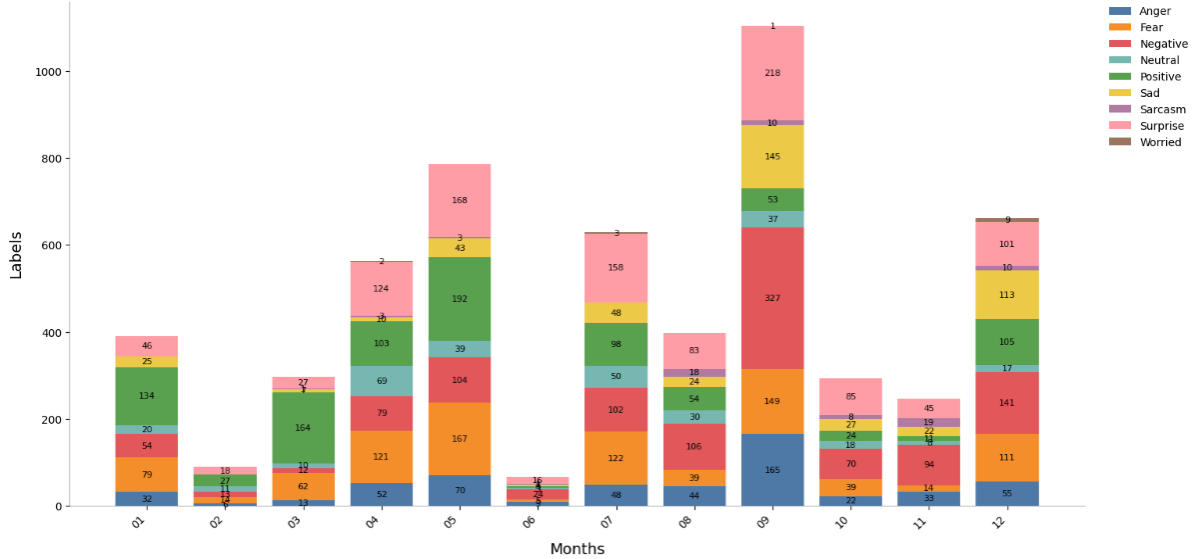
Monthly distribution of 9 emotional labels on the Weibo Platform (2024-2025). This stacked bar chart presents the absolute monthly frequency and proportional distribution of the 9 classified emotional tags.

**Figure 7:**
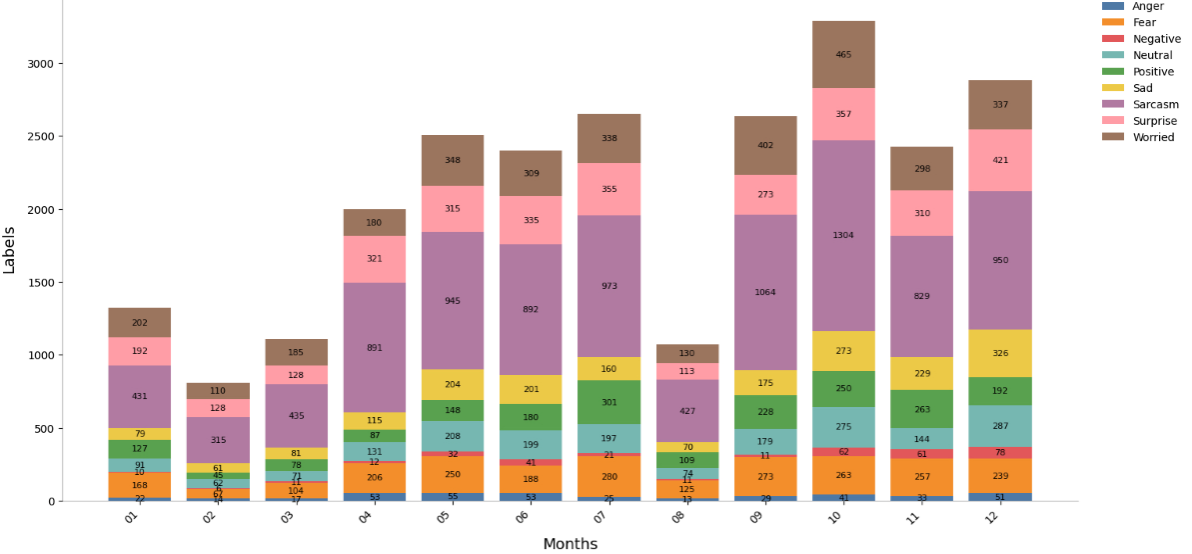
Monthly distribution of 9 emotional labels on the X (formerly Twitter) platform (2024-2025). This stacked bar chart displays the absolute monthly frequency and proportional distribution of the 9 classified emotional tags.

To visually illustrate the emotional distribution patterns of user comments, Figures 6 and 7 illustrate the monthly emotional distribution patterns for both platforms from 2024 to 2025. Platform X (formerly Twitter) exhibited a significantly higher comment volume; from an initial collection of 19,813 entries, the final analyzed sample for Platform X consisted of 21,600 emotion labels (mean 1800, SD 601.78). Similarly, the Weibo dataset was refined from an original 6536 entries to a final sample of 5527 emotion labels (mean 460, SD 223.13). The distribution of emotional labels on each platform showed certain differences (as shown in Table 2). “Fear” (16.68%) and “Negative” (20.37%) emotions were most prominent on Weibo, while “Sarcasm” (43.49%) dominated on X (formerly Twitter). As the emotion classification was derived from linguistic patterns without full contextual interpretation, the results should be viewed as indicative rather than absolute, particularly for context-dependent emotions such as sarcasm. Overall, the distribution reveals clear cross-platform differences, suggesting culturally distinct emotional response patterns during crises.

On Weibo (Figure 6), negative emotions such as “Fear” and “Worried” rise sharply in crisis months such as July and November. This pattern matches China’s high uncertainty avoidance tendency. Data from X (formerly Twitter; Figure 7) show a more stable distribution of these emotions (coefficient of variation [CV]=0.39), reflecting higher uncertainty tolerance in individualistic cultures with low avoidance traits. Emotional responses on Weibo often evolve into collective worry; X (formerly Twitter) users, however, express emotions more individually.

“Positive” emotions (such as prayers and gratitude) on Weibo increase sharply during disasters. For example, there were 192 labels in May and 105 in December. This pattern matches collectivist cultures’ focus on unity. On platform X (formerly Twitter), “Positive” emotions show a more even distribution (CV=0.51), suggesting higher emotional self-regulation and less need for group comfort.

“Sarcasm” shows the greatest difference. On platform X (formerly Twitter), sarcasm occurs often (n=9393, 43.49%; 95% CI 42.8%-44.2%). It spreads evenly across the year (CV=0.39) and reaches the highest level during emergencies, usually targeting institutional issues. This fits cultures with low power distance and strong free expression norms. On Weibo, sarcasm is rare (n=76), mainly appearing when disaster response is clearly poor. This reflects the caution typical of high power distance and collectivist environments.

“Surprise” appears on both platforms (Weibo: n=1089 and X [formerly Twitter]: n=1913), but the tone differs. On X (formerly Twitter), people explicitly show surprise at event scale or shock, matching direct cultural styles. On Weibo, surprise is brief, quickly turning into emotions such as sympathy or worry. This fits the pattern of emotional control in high-uncertainty-avoidance cultures. These emotional patterns across platforms highlight how culture influences not only the frequency but also the style and timing of public emotional expression in response to emergencies.

**Table 2:** The overall emotional distribution comparison between Weibo and X (formerly Twitter).

Tags	Weibo count	Value, % (95% CI)	X (formerly Twitter) count	Value, % (95% CI)	Total count	Value, % (95% CI)	OR^a^ (95% CI)
Anger	549	9.93 (9.1-10.7)	406	1.88 (1.7-2.1)	955	3.52 (3.3-3.7)	5.76 (5.04-6.57)
Fear	922	16.68 (15.7-17.7)	2252	10.43 (10.0-10.8)	3174	11.7 (11.3-12.1)	1.72 (1.58-1.87)
Negative	1126	20.37 (19.3-21.4)	345	1.60 (1.4-1.8)	1471	5.42 (5.2-5.7)	15.76 (13.91-17.85)
Neutral	313	5.66 (5.1-6.3)	1918	8.88 (8.5-9.3)	2231	8.22 (7.9-8.6)	0.62 (0.54-0.70)
Positive	967	17.5 (16.5-18.5)	2008	9.30 (8.9-9.7)	2975	10.97 (10.6-11.3)	2.07 (1.90-2.25)
Sad	467	8.45 (7.7-9.2)	1974	9.14 (8.8-9.5)	2441	9.00 (8.7-9.3)	0.92 (0.83–1.02)
Sarcasm	76	1.37 (1.1-1.7)	9393	43.49 (42.8-44.2)	9469	34.91 (34.3-35.3)	0.02 (0.01-0.02)
Surprise	1089	19.7 (18.7-20.8)	1913	8.86 (8.5-9.2)	3002	11.07 (10.7-11.4)	2.53 (2.33-2.74)
Worried	18	0.33 (0.2-0.5)	3304	15.3 (14.8-15.8)	3322	12.25 (11.9-12.6)	0.02 (0.01-0.03)
Total	5527	—	21600	—	27127	—	—

^a^R: odds ratio; calculated with X (formerly Twitter) as the reference group. All OR values include leading zeros for values <1 per journal requirements.

### Discovery of Online Public Opinion Topics

In the digital age, understanding the main topics in online public discourse during emergencies is crucial for understanding public attention, concerns, and information dissemination patterns. This section explores the dominant themes that appear on the 2 social media platforms in relation to emergency events. It uses c-TF-IDF to identify and rank key topics.

**Figure 8:**
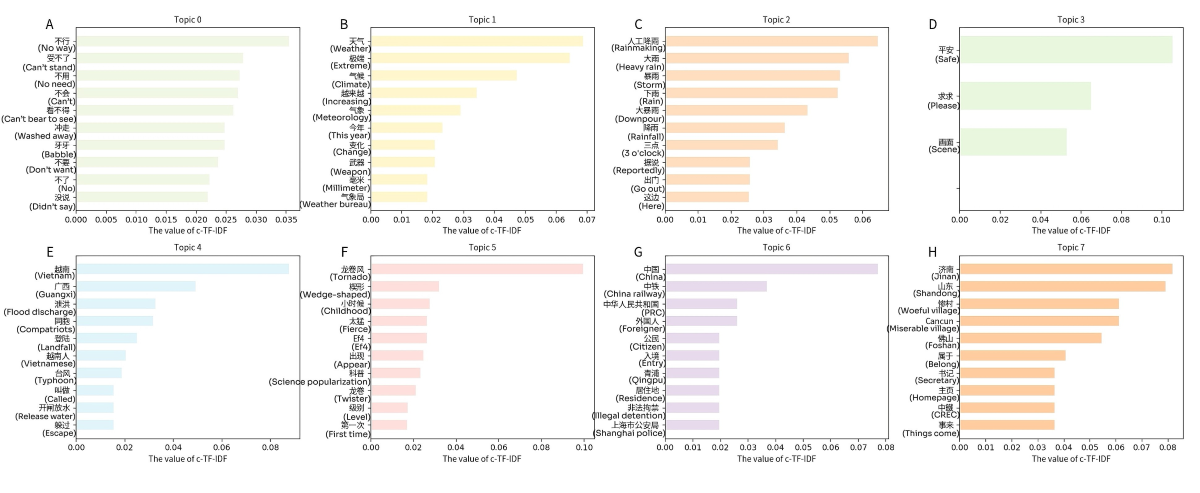
Dominant themes and ranked keywords on the Weibo platform, identified by class-based term frequency-inverse document frequency (c-TF-IDF) (Topics A-H). This figure displays the top 8 (of 345 total) extracted themes, highlighting the most representative Chinese tokens and their c-TF-IDF scores.

**Figure 9:**
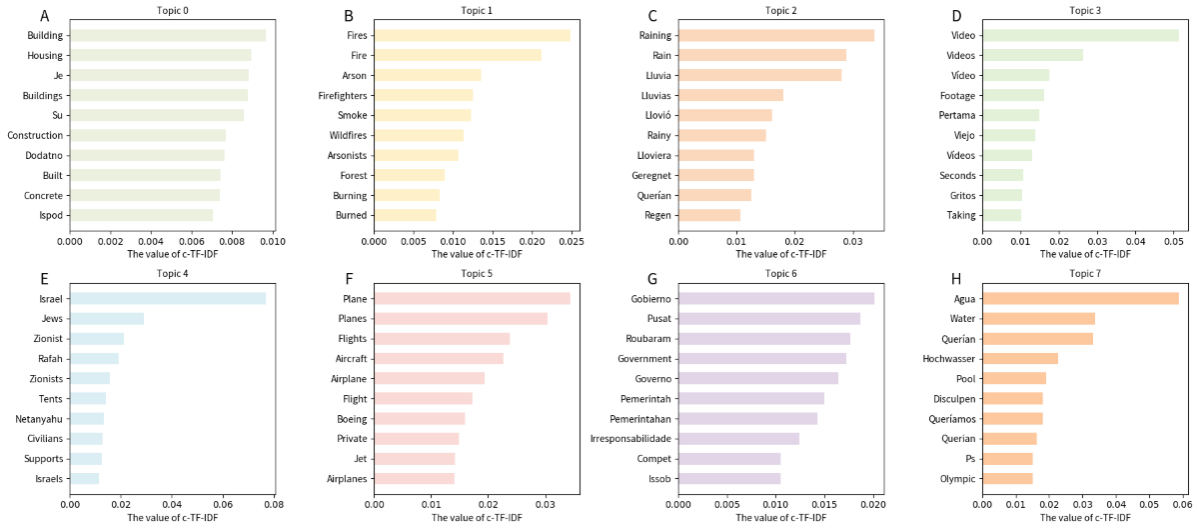
Dominant themes and ranked keywords on the X (formerly Twitter) platform, identified by class-based term frequency-inverse document frequency (c-TF-IDF) (Topics A-H). This figure presents the top 8 (of 1071 total) extracted themes, showing the highest-ranking tokens and their c-TF-IDF scores.

Figures 8 and 9 display the top 8 themes on Weibo and X (formerly Twitter), respectively. A total of 1071 topics were calculated for X (formerly Twitter), and 345 for Weibo. It can be observed that on both platforms, keywords directly related to emergency events (such as “rainstorm,” “hurricane,” “fire,” “airplane,” “death,” and so on) occupy significant positions. Regardless of cultural background, the public generally shows instinctive concern for disaster impacts when faced with real-world problems such as threats to life and property loss. Both platforms focus heavily on discussions about natural disasters (earthquakes, floods, typhoons, and so on). Characteristic words such as “earthquake,” “rainstorm” (on Weibo),” “hurricane,” “storm,” and “natural disasters” (on X [formerly Twitter]) are all high-frequency themes. This reflects that emergency events attract widespread attention across cultures, consistent with humans’ instinctive responses to survival and safety.

For comparative analysis, this study focused on the 7 most frequent topics within a subset of clusters from Weibo and X (formerly Twitter). For each of these topics, the 10 most frequent topic words were extracted (refer to Tables 3 and 4). The analysis identified representative themes, including “Rainfall impact” and “Fire crisis.”

**Figure 10:**
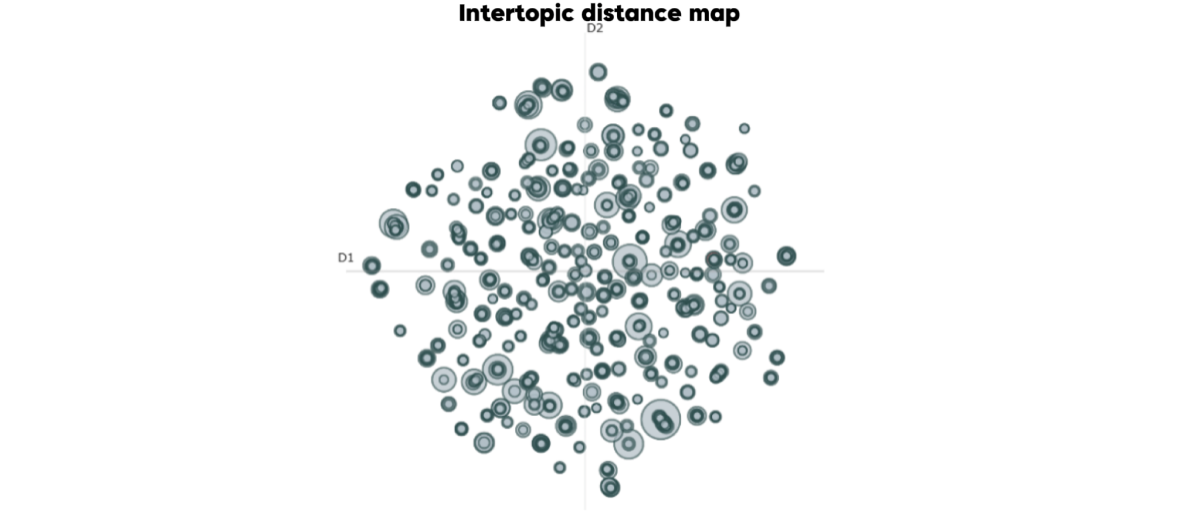
Bidirectional Encoder Representations from Transformers Topic (BERTopic) intertopic distance map visualizing the semantic distribution and prevalence of topics on Weibo. This map uses UMAP-based dimensionality reduction to plot topics, where the size of each circle corresponds to the topic frequency. The proximity of the circles illustrates semantic similarity. The overall visualization confirms that public discourse on Weibo is thematically concentrated.

**Figure 11:**
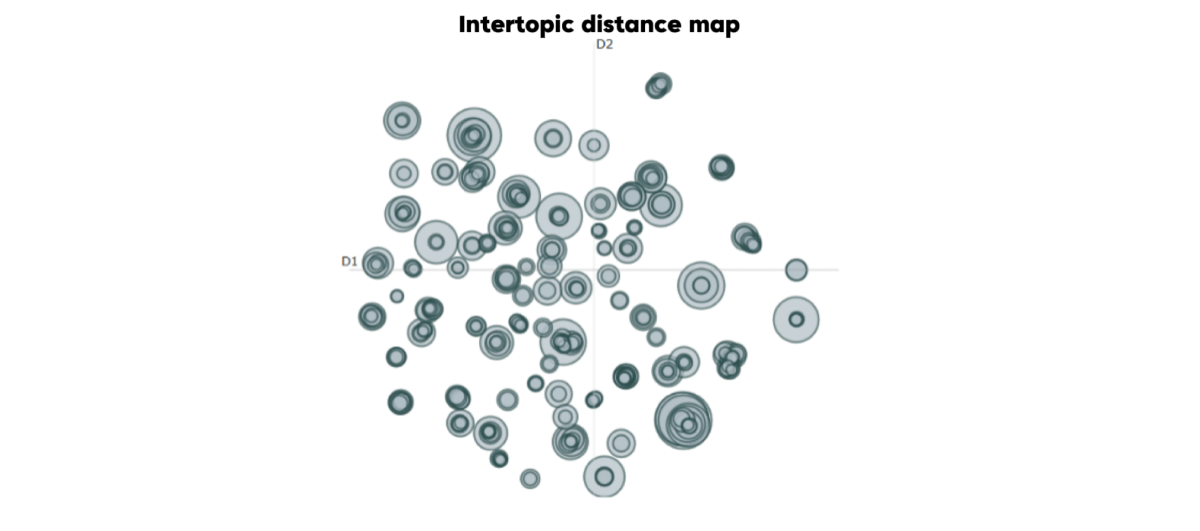
Bidirectional Encoder Representations from Transformers Topic (BERTopic) intertopic distance map visualizing the semantic distribution and prevalence of topics on X (formerly Twitter). This map displays the relative distance and size (topic prevalence) of topics derived from the BERTopic model. The broad spread of the circles across the map confirms that public discourse on X is thematically dispersed and covers a wider range of distinct issues than Weibo.

These were further examined using intertopic distance maps (Figures 10 and 11), which visualize topic prevalence and semantic similarity, revealing that discussions on Weibo were more concentrated, while those on X were more dispersed.

**Table 3:** The 7 most frequent topics and top keywords within selected clusters on the Weibo platform. This table presents the top 7 themes and their 10 highest-weighted keywords (based on class-based term frequency-inverse document frequency [c-TF-IDF] scores) that dominate public discourse on Weibo regarding emergency events.

Keywords		Weights
**Topic 0**		
	城市(City)	0.03
	危险(Danger)	0.07
	气象(Meteorological)	0.02
	我们(We)	0.01
	越南(Vietnam)	0.11
	猴子(Monkey)	0.02
	他们(They)	0.05
	打开(Open)	0.03
	三浦(Sanpu)	0.04
	这样(This way)	0.03
**Topic 1**		
	下雨(Raining)	0.06
	三点(Three o’clock)	0.05
	暴雨(Heavy rain)	0.04
	出门(Go out)	0.07
	这边(Here)	0.03
	大雨(Heavy rain)	0.06
	凌晨(Early morning)	0.4
	下雨天(Rainy day)	0.3
	平安(Safety)	0.22
	今晚(Tonight)	0.12
**Topic 2**		
	人工降雨(Artificial rain)	0.04
	大雨(Heavy rain)	0.06
	大暴雨(Torrential rain)	0.04
	降雨(Rainfall)	0.02
	太多了(Too much)	0.02
	强暴雨(Severe rainstorm)	0.46
	吓人(Frightening)	0.85
	平安(Safety)	0.22
	顺利(Smoothly)	0.11
	雨势(Rain intensity)	0.12
**Topic 3**		
	洪灾(Floods)	0.03
	遭遇(Encounter)	0.03
	中东地区(Middle East)	0.02
	东非(East Africa)	0.03
	上古(Ancient times)	0.01
	旱灾(Drought)	0.31
	防灾(Disaster prevention)	0.03
	失踪(Missing)	0.08
	甘蔗(Sugarcane)	0.02
	极端(Extreme)	0.09
**Topic 4**		
	火之歌(A Song of Ice and Fire)	0.07
	火烧(Burning)	0.04
	起火(Catch fire)	0.04
	大火(Big fire)	0.04
	火灾(Fire disaster)	0.04
	纵火(Arson)	0.03
	烟头(Cigarette butt)	0.02
	烟火(Fireworks)	0.02
	绝好(Excellent)	0.02
	素材(Material)	0.01
**Table 5**		
	山火(Forest fire)	0.08
	扑灭(Extinguish)	0.05
	肆虐(Raging)	0.04
	尽快(As soon as possible)	0.04
	火灾(Fire disaster)	0.04
	救火(Firefighting)	0.03
	见证(Witness)	0.02
	菲政府(Philippine government)	0.01
	关爱(Care)	0.03
	减轻(Alleviate)	0.02
**Topic 6**		
	祈祷(Pray)	0.32
	人们(People)	0.05
	保守(Conservative)	0.04
	无人(No one)	0.8
	恳求(Plead)	0.75
	祈祷平安(Pray for safety)	0.17
	阿弥陀佛祈祷(Amitabha prayer)	0.8
	生活(Life)	0.04
	活着(Alive)	0.04
	大过年(During Chinese New Year)	0.11

**Table 4:** The 7 most frequent topics and top keywords within selected clusters on the X (formerly Twitter) platform. This table outlines the top 7 themes and their 10 highest-weighted keywords (based on class-based term frequency-inverse document frequency [c-TF-IDF] scores) identified in the X discourse on emergency events.

Keywords			Weights
**Topic 0**			
	Fires	0.08	
	Fire	0.07	
	Arson	0.04	
	Smoke	0.3	
	Firefighters	0.03	
	Arsonists	0.04	
	Burning	0.02	
	Burned	0.01	
	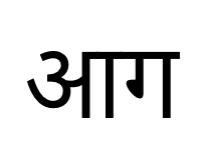 (Fire)	0.04	
	Flames	0.19	
**Topic 1**			
	Gas	0.11	
	Fuel	0.3	
	Residential	0.14	
	Tanker	0.28	
	Cylinder	0.04	
	Station	0.03	
	Lorry	0.05	
	Embakasi	0.07	
	Plant	0.03	
	Cylinders	0.05	
**Topic 2**			
	Portugal	0.14	
	Wildfire	0.05	
	Fires	0.05	
	Wildfires	0.04	
	Spanish	0.04	
	Portugals	0.04	
	Portuguese	0.04	
	Declaran	0.02	
	Alguém	0.03	
	Arson	0.04	
**Topic 3**			
	Lava	0.07	
	Lewotobi	0.08	
	Lakilaki	0.05	
	Mount	0.09	
	Ash	0.07	
	Indonesia	0.15	
	Evacuations	0.07	
	Erupted	0.07	
	Eruptions	0.06	
	16000	0.04	
**Topic 4**			
	Bus	0.17	
	Thailand	0.11	
	44	0.77	
	Children	0.13	
	Returning	0.08	
	Siswa	0.05	
	Guru	0.03	
	Bangkok	0.07	
	Teachers	0.11	
	Thani	0.02	
**Topic 5**			
	Gobierno	0.25	
	Government	0.07	
	Gobierna	0.12	
	Governo	0.08	
	Pemerintah	0.09	
	Federal	0.03	
	Pusat	0.06	
	Roubaram	0.05	
	Años	0.05	
	Provincias	0.08	
**Topic 6**			
	Rescue	0.16	
	Underway	0.03	
	Rescued	0.13	
	Certain	0.05	
	Humourrescue	0.03	
	Baseball	0.02	
	Operationswhat	0.02	
	Isvbeing	0.03	
	Resgate	0.05	
	Missions	0.1	

The intertopic distance map for X (formerly Twitter; Figure 11) reveals a relatively dispersed topic structure, suggesting that emergency event discussions cover a wide range of issues. A key trait of its discourse is the coexistence of several distinct clusters alongside some relatively isolated topics. For example, one prominent cluster centered on the immediate impact and characteristics of specific disasters, particularly those related to weather events. As shown in Table 4, this cluster covers Topic 0 (“fire, arson, smoke...”), with a count of 198; Topic 1 (“gas, fuel, residential, tanker”), with a count of 99; and Topic 2 (“portugal, wildfire, fires, wildfires, alguém”), with a count of 30, as evidenced by their proximity in the map and shared keywords. These topics highlight discussions about sudden natural disaster events and the dangers they pose to affected urban areas. Another cluster appears to revolve around the broader disaster context, including Topic 3 (“lava, lewotobi, lakilaki, mount, ash...”), with a count of 23, and Topic 4 (“bus, Thailand, children, returning, siswa...”), with a count of 17. While these topics are distinct, they all focus on sudden fire-related events. Simultaneously, a corresponding yet somewhat independent cluster emerges. It includes Topic 5 (“gobierno, government, gobierna, governo, pemerintah, federal, pusat, roubaram, años, provincias”), with a count of 167. Topic 6 (“rescue, underway, rescued, baseball, operations, isvbeing, resgate, missions”), with a count of 11, stands out as a major theme, indicating a strong emphasis on rescue efforts and on-the-scene situations during emergencies. The topic including “earthquake, sismo, earthquakes, terremoto...” is also relatively large, with a count of 63, suggesting substantial discussion surrounding seismic events. Notably, distinct topics focusing on long-term recovery or preventive measures are relatively scarce, though these elements appear to some extent in earthquake and flood themes.

In contrast, as shown in Figure 10, the topic structure on Weibo is more concentrated, suggesting a stronger connection among themes. Similar to X (formerly Twitter), a significant cluster on Weibo pertains to natural disasters, but with a greater emphasis on direct impacts and local contexts. It is evident within Cluster 1 that Topic 0 from Table 3 (count=51), including “城市 (city),” “危险 (danger),” “气象 (meteorological),” “我们 (we),” “越南 (Vietnam),” “猴子 (monkey),” “他们 (they),” “打开 (open),” “三浦 (Sanpu),” and “这样 (this)”; Topic 1 (count=30), with terms such as “下雨 (raining),” “三点 (3 o’clock),” “暴雨 (heavy rain),” “出门 (go out),” “这边 (here),” “大雨 (heavy rain),” “凌晨 (early morning),” and “下雨天 (rainy day)”; Topic 2 (count=20), including “人工降雨 (artificial rain),” “大雨 (heavy rain),” “大暴雨 (torrential rain),” “降雨 (rainfall),” “太多了 (too much),” “强暴雨 (severe rainstorm),” “吓人 (frightening),” “平安 (peace),” and “顺利 (smoothly)”; and Topic 3 (count=7), with “洪灾 (floods),” “遭遇 (encounter),” “中东地区 (Middle East),” “东非 (East Africa),” “上古 (ancient times),” “旱灾 (drought),” and “边境县 (border county),” are closely located on the map and share keywords related to weather events and urban impacts. Another cluster encompasses similar natural disaster themes, such as Topic 4, including “火之歌 (A Song of Ice and Fire),” “火烧 (burning),” “起火 (catch fire),” “大火 (big fire),” “火灾 (fire disaster),” “纵火 (arson),” “烟头 (cigarette butt),” and “烟火 (fireworks)”; and Topic 5, including “山火 (forest fire),” “扑灭 (extinguish),” “肆虐 (raging),” “尽快 (as soon as possible),” “火灾 (fire disaster),” “救火 (firefighting),” “见证 (witness),” “政府 (government),” “关爱 (care),” and “减轻 (alleviate).” Although different from the rainfall themes, these topics still fall under the broader category of natural disasters. Similarly, themes such as Topic 6 (count=24) are more mixed, including “祈祷 (pray),” “人们 (people),” “保守 (conservative),” “无人 (no one),” “恳求 (plead),” “祈祷平安 (pray for peace),” “阿弥陀佛祈祷 (Amitabha prayer),” and “生活 (life).”

Topic analysis uncovers a common trend. Both platforms place strong emphasis on the direct impact of natural disasters. This focus is supported by keyword clusters related to heavy rainfall, floods, and earthquakes. This shared focus reflects basic human reactions to the dangers posed by these events. Weibo users tend to focus more on the natural phenomena themselves, such as “暴雨 (heavy rain),” “气候变暖 (climate warming),” and “极端天气 (extreme weather).” Their comments primarily describe disaster severity and meteorological anomalies, with emotions dominated by “担忧 (worry)” and “无奈 (helplessness).” In contrast, X (formerly Twitter) users often connect “rainfall” or “storm” to climate change or government action, expressing more explicit criticism. This difference corresponds to Hofstede’s cultural dimensions theory. In highly collectivist societies (Weibo), discourse emphasizes shared communal emotions; in low collectivist contexts (X [formerly Twitter]), it prioritizes individual critique and responsibility attribution.

Under the “Fire crisis” theme, Weibo comments frequently feature keywords such as “消防员 (firefighters),” “肆虐 (raging),” and “扑灭 (extinguish),” expressing respect for firefighters and concern about the spread of disasters. Emotions here are concentrated in “fear” and “worried.” On X (formerly Twitter), fire-related discussions include sympathy for victims, along with more anger toward arsonists and questions about the adequacy of firefighting resources. This phenomenon illustrates how people in high uncertainty avoidance cultures tend to simplify perceived causes of disasters. It also shows how the public in low power distance cultures more directly criticizes institutional failures.

For the “Air crash” theme, Weibo users primarily express “震惊 (surprise)” and “悲痛 (sadness),” using keywords such as “飞机 (airplane),” “故障 (malfunction),” “失控 (out of control),” and “逝者 (the deceased).” This reflects emotional responses and a tendency toward collective mourning when facing uncontrollable disasters. X (formerly Twitter) comments, by contrast, more frequently focus on flight safety standards and airline management, adopting a calmer and more rational tone. This pattern underscores the emphasis on factual accuracy and responsibility in highly individualistic cultural contexts. The theme also includes some sarcastic remarks on X (formerly Twitter), such as comments mocking the slowness of accident investigations, which are barely present on Weibo.

This study shows how emotional expressions and narrative focus differ across cultures. It introduces an “emotion-culture mapping table” (Table 5). The table compares user responses to emergency topics on Weibo and X (formerly Twitter). It also links these response patterns to Hofstede’s cultural dimensions. In addressing possible subjective interpretation, especially when linking emotions and themes to cultural dimensions in Table 5, a verification step was added. A subset of comments was independently analyzed by 2 human coders to confirm the categorization principles in the emotion-culture mapping table. The intercoder reliability test, measured by Cohen κ, showed substantial agreement (κ=0.82). This validation step ensures that the interpretations in Table 5 are not isolated but are systematic and empirically supported by a reliable coding method.

The table outlines 6 cultural dimensions derived from 4 core theories (such as high or low power distance). For each dimension, it details the corresponding “Narrative Focus Examples” and provides relevant “Dimension explanation and link,” thus clearly constructing the mapping relationship between culture and public narratives.

**Table 5:** The emotion-culture mapping table provides for a clearer understanding of emotions within the framework of Hofstede’s cultural dimension theory. This table systematically compares the observed dominant emotions and narrative focus on the 2 platforms and provides a theoretical explanation for observed cross-cultural variations in online discourse regarding emergency events.

Hofstede dimension	Cultural context	Platform	Dominant emotions	Narrative focus examples	Dimension explanation and link
Power distance	Low power distance	X (formerly Twitter)	Anger and sarcasm	Direct criticism of institutional failures.	Low power distance cultures tend to question authority and power structures, and the public is more likely to directly criticize institutional failures.
Power distance	High power distance	Weibo	Fear and worry	Respect for firefighters and concern about the disaster spread.	High power distance cultures tend to respect authority, focus more on collective emotions, and exhibit less criticism of institutions.
Uncertainty avoidance	High uncertainty avoidance	Weibo	Fear and worry	Concern about the disaster spread.	High uncertainty avoidance cultures tend to seek certainty, and explanations of disaster causes may be simplified, with a focus on immediate threats.
Uncertainty avoidance	Low uncertainty avoidance	X (formerly Twitter)	Anger and sarcasm	Anger toward arson and questioning the adequacy of firefighting resources.	Low uncertainty avoidance cultures are more tolerant of uncertainty and tend to explore complex disaster causes, including human and institutional factors.
Individualism	High individualism	X (formerly Twitter)	Anger, sarcasm, and sad	Discussion of flight safety standards, airline management, a strong focus on factual truth, and responsibility attribution.	High individualistic cultures emphasize personal responsibility and autonomy, focusing on factual truth, responsibility attribution, and rational analysis.
Individualism	Low individualism	Weibo	Fear, surprise, and positive (pray and wish)	Emotional outburst and collective mourning.	Low individualistic cultures emphasize group cohesion and collective emotions, and tend to express emotions communally, such as through collective mourning.
Collectivism	High collectivism	Weibo	Worried, fear, and positive	Description of disaster intensity, meteorological anomalies, and sharing of communal emotions.	High collectivist cultures emphasize group connections and shared feelings, and tend to share common worries and helplessness, with a focus on natural phenomena.
Collectivism	Low collectivism	X (formerly Twitter)	Sarcasm	Linking rainfall to climate change and government responses, individual criticism, and responsibility attribution.	Low collectivist cultures tend toward independent thinking and critical analysis, focusing on the underlying causes of events and responsibility.

**Figure 12:**
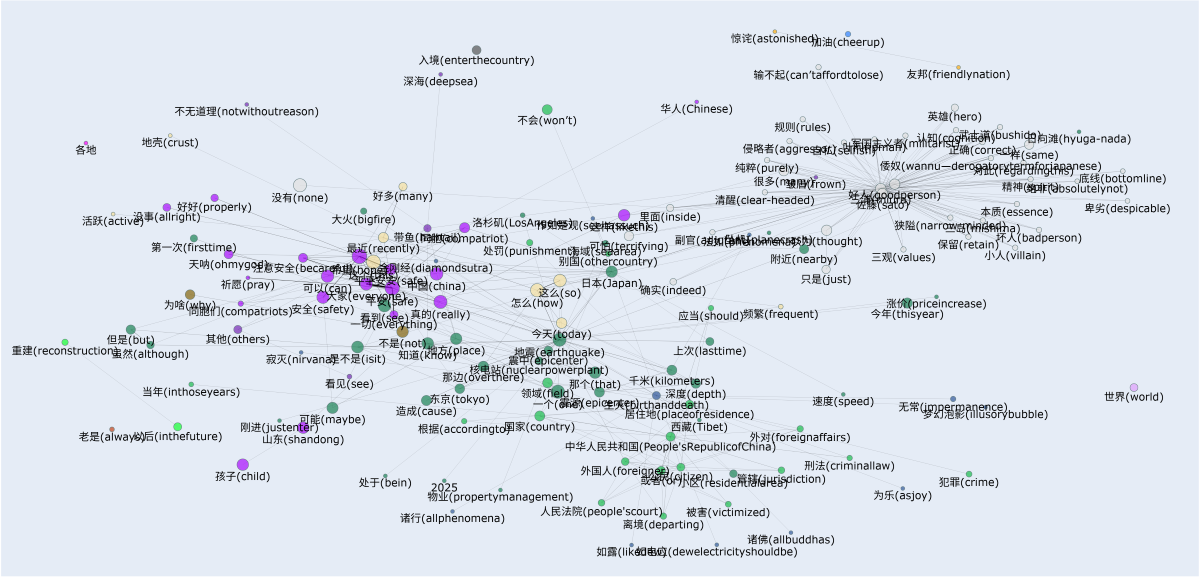
Semantic co-occurrence network graph of top terms on the Weibo platform. The network visualizes the thematic connections and semantic cores in the public discourse, constructed from the top 155 most frequent terms. Node size represents word frequency, edge thickness indicates co-occurrence strength, and colors delineate distinct thematic clusters.

**Figure 13:**
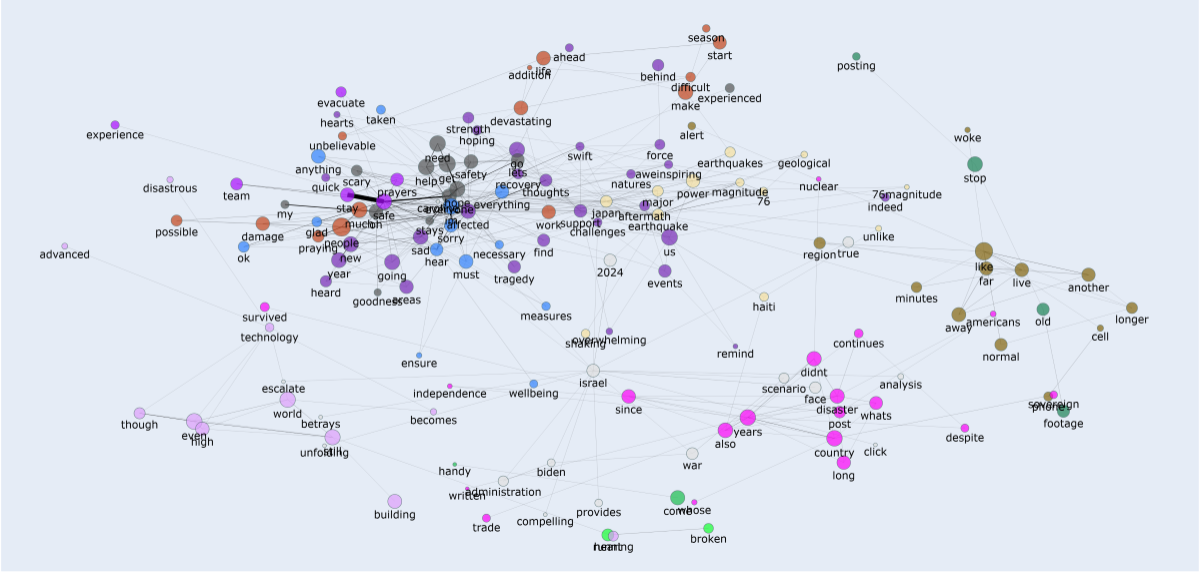
Semantic co-occurrence network graph of top terms on the X (formerly Twitter) platform. The network visualizes the thematic connections and semantic cores in the public discourse, constructed from the top 155 most frequent terms. Node size represents word frequency, the thickness of the edge indicates the co-occurrence strength, and colors denote thematic clusters.

Semantic co-occurrence network graphs (Figures 12 and 13) were constructed based on word adjacency data (the top 155 terms). In the graphs, node size indicated word frequency, edge thickness represented co-occurrence strength, and colors denoted semantic communities. This approach helps to reveal semantic cores, thematic connections between clusters, and cultural differences in public discourse across platforms. Overall, the Weibo network displayed cohesive and centralized emotional narratives, whereas the X (formerly Twitter) network showed fragmented and decentralized discourse patterns. Unlike topic clustering (Figures 10 and 11), this method did not group texts by overall theme similarity; instead, it focused on word connections at a lexical level, providing a bottom-up perspective of how meaning clusters naturally formed in the discourse.

Take Weibo as an example. On this social platform, words such as “平安 (safety),” “祈祷 (pray),” “安置 (resettlement),” and “地方 (local)” created tight clusters. This finding suggests a strong narrative connection among emotional support, local identity, and institutional action. Conversely, semantic clusters on X (formerly Twitter; eg, “pray,” “rescue,” “fire,” and “government”) were more scattered. This pattern shows narratives that were emotional yet fragmented; instead of shaping a single shared story, multiple narrative threads exist side by side. High-frequency words found in topic clustering (eg, “fire” and “floods”) spread across different parts of the network. These words also appear together with various other keywords, highlighting emotional differences that topic models alone failed to capture. One specific case is “fire” on X (formerly Twitter). In 1 cluster, it appears alongside “arson” and “anger,” while in another cluster, it is linked to “rescue” and “prayers.” This example proves there were emotional complexities that a single topic label could not fully represent.

These patterns are consistent with the cultural differences discussed previously. The semantic cohesion observed in the Weibo graph suggests that collectivist cultures tend to build discourse around shared symbols and centralized values. In contrast, the scattered distribution of words on the X (formerly Twitter) platform implies a more decentralized response style, which is typical of individualistic cultures. Therefore, the semantic co-occurrence network analysis does not merely repeat the conclusions of topic clustering. Instead, it contributes to a deeper understanding of how users interconnected emotions, facts, and cultural expressions during emergencies.

## Discussion

### Principal Findings

This study provides empirical evidence that cultural values influence emotional expression and discourse during global emergencies, directly relating to the research goals stated in the introduction. The study used a large-scale dataset of comments from Weibo and X (formerly Twitter) (2024-2025) and identified significant cross-platform differences in both emotional distribution and topic structures. On Weibo, negative emotions such as fear and worry are more common, reflecting China’s high uncertainty avoidance. Collective expressions, including prayers and wishes for safety, are also frequent, reflecting collectivist orientations. By contrast, X (formerly Twitter) comments contain more sarcasm and criticism, reflecting individualistic values and lower power distance.​ Topic modeling supported these patterns: Weibo discourse focuses more on disaster severity and communal support, whereas X (formerly Twitter) discourse emphasizes accountability, institutional response, and climate-related narratives. These findings confirm that Hofstede’s cultural dimensions—including individualism, collectivism, power distance, and uncertainty avoidance—are useful for explaining cultural differences in online crisis communication.

### Comparison With Previous Work

The study supports earlier findings that cultural background strongly shapes emotional expression in crises. On Chinese platforms, collectivist traits dominate, as noted by Zhang [11], where prayers and calls for peace act as clear signs of group unity. This corresponds closely to the Weibo data analyzed in the research, where high-frequency expressions include keywords such as “pray” and “wish for safety.” These expressions demonstrate the tendency of collective emotions. From a Western context perspective, the sarcastic and individualized responses described by Imran et al [62] and Maynard and Greenwood [63] match the study’s observations. Sarcasm appeared widespread on X (formerly Twitter), often targeting institutional failures. Wang et al [7] also emphasized that cultural orientation affects how people attribute responsibility and process crisis-related information. The study extends this perspective: X (formerly Twitter), which has lower power distance, shows users openly criticizing authorities. On Weibo, however, higher power distance prevails, and emotions lean more toward worry and collective solidarity. The study by Matsumoto et al [64] emphasized the impact of cultural differences on emotional expression, yet it relied heavily on experimental and questionnaire data, with no inclusion of natural language evidence in the context of social media. Building on this foundation, this study helps fill the gap in empirical natural language research within this field, providing a new supporting dimension for the association between cultural differences and emotional expression.

The research also matches earlier studies when examining discourse structure and interaction styles. Han and Wang [65] and Chen and Yik [66] pointed out that Chinese users’ discourse tends to emphasize group harmony and shared emotions. This was confirmed by the topic and semantic network analysis in this study, which revealed cohesive clusters around words such as “pray” and “safety.” Discourse on X (formerly Twitter), by contrast, appeared more fragmented. Loosely connected clusters formed around terms such as “damage,” “government,” and “help,” a pattern consistent with Gu et al [67], who stressed the openness and diversity of Western crisis communication. In addition, previous research has shown that in low power distance cultures, Western publics are more likely to hold institutions directly accountable [6,68]. The study contributes to this body of work by demonstrating that sarcasm, specifically, acts as a discursive marker of individualistic cultural values. In addition, as noted in the study by Du et al [69], although Chinese participants scored slightly lower than American participants in understanding sarcasm, they pointed out that “collectivism” is instead associated with better sarcasm comprehension. However, understanding sarcasm does not equate to using sarcasm. This is influenced by cultural contexts and platform rules. Precisely due to this discrepancy, only a minimal amount of sarcasm was observed in the Weibo corpus analyzed in this study. This finding demonstrates the importance of this study’s focus on cultural dimensions. When these observations are taken together, the research both confirms and advances previous scholarship. It integrates Hofstede’s cultural dimensions with a multimethod analysis, and in doing so, provides a more comprehensive account of the culturally distinctive online responses observed.

### Limitations and Future Directions

This study has several limitations. First, the dataset was limited to 2 platforms, which, while influential, cannot fully represent the diversity of cultural and digital contexts. Future research should include additional platforms such as Reddit, YouTube, or regional networks to enhance representativeness. Second, cultural differences were inferred from platform-level data, which may risk oversimplifying individual identities and cross-cultural hybridity. Third, although XLM-RoBERTa and BERTopic provided robust classification, automated labeling relies on linguistic and semantic cues and does not fully incorporate contextual validation. Because of this, some detailed expressions, such as sarcasm, might be undercounted or labeled wrong. Manual validation and mixed methods approaches could strengthen accuracy in future studies. Finally, while the 2024-2025 time frame captured multiple emergencies, it did not account for the effects of event type or severity. Longer longitudinal studies across multiple regions would help clarify how cultural and contextual factors interact in shaping online discourse.

Although event scale, comment volume, and posting time were not the main focus, they could still indirectly influence the observed cross-cultural differences in emotion and discourse patterns.

### Conclusions

This study shows that digital emergency discourse is structured by culturally embedded values rather than operating as a neutral information space. Cross-platform analysis demonstrates systematic associations between cultural dimensions and patterns of emotional expression, narrative focus, and responsibility attribution. By operationalizing cultural dimensions through a mixed methods analysis of large-scale, multilingual, naturally occurring social media data, this study advances infodemiology scholarship from descriptive mapping of online content to theory-informed, mechanism-oriented analysis, beyond survey-based and single-platform approaches. It provides a replicable comparative framework for examining how cultural meaning systems are translated into observable digital traces under conditions of uncertainty, risk, and collective sense-making.

From an applied perspective, the findings offer concrete guidance for multiple stakeholders. For governments and public health authorities, the results highlight the need to tailor risk communication to cultural expectations by emphasizing reassurance, unity, and structured guidance in high–uncertainty-avoidance contexts, and transparency, responsiveness, and accountability in low–power-distance contexts. For international organizations, the findings suggest that global crisis messaging should prioritize culturally neutral framing, shared risk narratives, and locally adaptable templates to minimize misunderstanding across cultural boundaries. For digital platforms, culturally sensitive governance is essential, including rapid rumor suppression in high-anxiety environments and careful differentiation between legitimate public critique and harmful misinformation in low–power-distance contexts. Together, these implications support the design of more equitable, trustworthy, and context-aware infodemic management strategies for future public health and safety emergencies.

## Appendix

Multimedia Appendix 1 JARS (Journal Article Reporting Standards) reporting checklist.

## Abbreviations

BERTopic: Bidirectional Encoder Representations from Transformers Topic
c-TF-IDF: class-based term frequency-inverse document frequency
CV: coefficient of variation
IG: Integrated Gradients
ISO: International Organization for Standardization
JARS: Journal Article Reporting Standards
XLM-RoBERTa: Cross-lingual Language Model–Robustly optimized BERT approach

## Data Availability

The datasets generated or analyzed during this study are available from the corresponding author on reasonable request.
