# Supplementary material for: Public Emotional and Thematic Responses to Major Emergencies on Social Media, 2024-2025: Cross-Sectional Convergent Mixed Methods Study
Source: J Med Internet Res. 2026 Jan 20;28:e84648. doi: 10.2196/84648 (PMC12869147; doi:10.2196/84648)
Supplement: Multimedia Appendix 1 [file jmir_v28i1e84648_app1.docx]

| JARS Section & Item | Detailed Requirement Description | Location in Manuscript (Para. No. / Section) |
| --- | --- | --- |
| 1. TITLE AND ABSTRACT |  |  |
| 1.1 Title | Identify the study as mixed methods and state the main variables/phenomena. | Para1-3 |
| 1.2 Mixed Methods Design | Specify the design type (e.g., convergent design) in the abstract. | Para12 |
| 1.3 Objectives | State the problem/question/objectives under investigation. | Para 8-11 |
| 1.4 Study design | Indicate the study design, including types of participants or data sources, analytic strategy | Para 12-20 |
| 1.5 Findings | Report findings, including effect sizes and confidence intervals or statistical significance levels. | Para 21-30 |
| 1.6 Conclusions | State conclusions, beyond just results, and report the implications or applications. | Para 31-37 |
| 2. INTRODUCTION |  |  |
| 2.1 Review of Relevant Scholarship | Provide a succinct review of relevant scholarship, including  ‒ relation to previous work  ‒ differences between the current report and earlier reports if some aspects of this study have been reported on previously | Para 80-166  Para 168-177 |
| 2.1 Research Problem | Frame the problem and explain why both quant and qual data are needed. | Para 170-172 |
| 2.2 Study Objectives | State specific hypotheses, aims, and objectives, | Para 175-186 |
| 3. METHOD - RESEARCH DESIGN |  |  |
| 3.1 Design Overview | Provide the rationale for using mixed methods and define the specific design used. | Para 189-200 |
| 3.2 Sequence & Priority | Describe the timing of the strands (parallel/sequential) and their relative importance. | Para 194-195 |
| 4. METHOD - QUANTITATIVE STRAND |  |  |
| 4.1 Inclusion and Exclusion | Report inclusion and exclusion criteria, including any restrictions based on demographic characteristics. | Inclusion: Para 205, 206  Exclusion: Para 208, 238 |
| 4.2 Participant Characteristics | Report major demographic characteristics | Not Applicable. The study analyzed publicly available text data. No individual user characteristics (e.g., age, sex, ethnicity, socioeconomic status) were accessed |
| 4.3 Sampling Procedures | Describe procedures for selecting participants | Para 204-207 |
|  | Describe settings and locations where data were collected as well as dates of data collection. | Para 200-201 |
|  | Describe agreements and payments made to participants. | Not Applicable. The data analyzed were publicly accessible, anonymized social media texts. |
|  | Describe institutional review board agreements, ethical standards met, and safety monitoring. | Para 343-355 |
| 4.3 Sample Size, Power, and Precision | Describe the sample size, power, and precision, including  ‒intended sample size  ‒achieved sample size, if different from the intended sample size  ‒determination of sample size, including  ›power analysis, or methods used to determine precision of parameter estimates  ›explanation of any interim analyses and stopping rules employed | intended sample size, para 204-205;  achieved sample size, para 220-222;  determination of sample size, para208;  No interim analyses or stopping rules were employed. Data collection was fixed between the predetermined start and end dates. |
| 4.4 Measures and Covariates | Define all primary and secondary measures and covariates, including measures collected but not included in the report. | Para 224-234 |
| 4.5 Data Collection | Describe methods used to collect data | Para 203-222 |
| 4.6 Quality of Measurements | Describe methods used to enhance the quality of measurements, including  ‒training and reliability of data collectors  ‒use of multiple observations | Para 225, 257-258, 287-304 |
| 4.7 Instrumentation | Provide information on validated or ad hoc instruments created for individual studies, for individual studies (e.g., psychometric and biometric properties). | Para 249-266 |
| 4.8 Masking | Report whether participants, those administering the experimental manipulations, and those assessing the outcomes were aware of condition assignments.  • If masking took place, provide a statement regarding how it was accomplished and whether and how the success of masking was evaluated. | Not Applicable. This was an observational computational study. Neither the data nor the researchers were masked to the source platform |
| 4.9 Psychometrics | Estimate and report values of reliability coefficients for the scores analyzed (i.e., the researcher’s sample);  Report estimates related to the reliability of measures; Report the basic demographic characteristics of other samples if reporting reliability or validity coefficients from those samples, such as those described in test manuals or in norming information for the instrument. | Para 265-271;  Internal Consistency: Not Applicable.as the measures are discrete classifications and not composite scales. |
| 4.10 Conditions and Design | State whether conditions were manipulated or naturally observed. Report the type of design as per the JARS–Quant tables | Para 203 |
| 4.11 Data Diagnostics | Describe planned data diagnostics, including  ‒criteria for post-data-collection exclusion of participants, if any  ‒criteria for deciding when to infer missing data and methods used for imputation of missing data  ‒definition and processing of statistical outliers  ‒analyses of data distributions  ‒data transformations to be used, if any | Para 206-207, 238-242 |
| 4.12 Analytic Strategy | Describe the analytic strategy for inferential statistics and protection against experiment wise error for  ‒ primary hypotheses  ‒ secondary hypotheses  ‒ exploratory hypotheses | Para 281-285 |
| 5. METHOD - QUALITATIVE STRAND |  |  |
| 5.1 Researcher Description | Describe the researchers' background and manage prior understandings. | Para 307-316 |
| 5.2Participants or Other Data Sources | •Provide the numbers of participants/documents/events analyzed.  • Describe the demographics/cultural information, perspectives of participants, or characteristics of data sources that might influence the data collected.  • Describe existing data sources, if relevant (e.g., newspapers, internet, archive).  • Provide data repository information for openly shared data, if applicable.  • Describe archival searches or process of locating data for analyses, if applicable. | Para 307-316 |
| 5.2 Researcher–Participant Relationship | • Describe the relationships and interactions between researchers and participants relevant to the research process and any impact on the research process (e.g., was there a relationship prior to research, are there any ethical considerations relevant to prior relationships) | Not Applicable. The data sources were anonymized, publicly available social media comments. There was no direct interaction or prior relationship between the researchers and the users who posted the comments. |
| 5.3 Participant Recruitment | Describe the recruitment process (e.g., face-to-face, telephone, mail, email) and any recruitment protocols. | Not Applicable. The data were collected from existing social media archives; no participants were recruited. |
| 5.4 Participant Selection | Describe the participants/data source selection process (e.g., purposive sampling methods, such as maximum variation; convenience sampling methods, such as snowball selection; theoretical sampling; diversity sampling) and inclusion/exclusion criteria.  • Provide the general context for the study (when data were collected, sites of data collection).  • If your participant selection is from an archived data set, describe the recruitment and selection process from that data set as well as any decisions in selecting sets of participants from that data set. | Para 307-310 |
| 5.5 Data Collection | State the form of data collected (e.g., interviews, questionnaires, media, observation). Describe the origins or evolution of the data-collection protocol. | Para 307-310 |
| 5.6 Recording and Data Transformation | Identify data audio/visual recording methods, field notes, or transcription processes used. | No audio or visual recording, field notes, or transcription processes were involved in this study. The qualitative component analyzed pre-existing written social media comments that were natively available in textual form. |
| 6. FINDINGS / RESULTS |  |  |
| 6.1 Participant Flow | Report the flow of participants, including  ‒ total number of participants in each group at each stage of the study  ‒ flow of participants through each stage of the study (include figure depicting flow, when possible | Para 386-388 |
| 6.2 Recruitment | Provide dates defining the periods of recruitment and repeated measures or follow-up. | Para 386 |
| 6.3 Statistics and Data Analysis | Provide information detailing the statistical and data-analytic methods used, including  ‒ missing data  ‒ descriptions of each primary and secondary outcome, including the total sample and each subgroup, that includes the number of cases, cell means, standard deviations, and other measures that characterize the data used  ‒ inferential statistics  ‒ complex data analyses  ‒ estimation problems  ‒ other data analyses performed  ‒ Report any problems with statistical assumptions and/or data distributions that could affect the validity of findings. | Since there are no missing data in the final analytical dataset, methods like the Little’s MCAR test and multiple imputations were not applicable. |
| 6.4 Results Subsections | Describe research findings (e.g., themes, categories, narratives) and the meaning and understandings that the researcher has derived from the data analysis. • Demonstrate the analytic process of reaching findings (e.g., quotes, excerpts of data). • Present research findings in a way that is compatible with the study design. • Present synthesizing illustrations (e.g., diagrams, tables, models), if useful in organizing and conveying findings. Photographs or links to videos can be used. | Para 384-553 |
| 7. DISCUSSION |  |  |
| 7.1 Support of Original Hypotheses | Provide a statement of support or nonsupport for all hypotheses, whether primary or secondary, including  ‒ distinction by primary and secondary hypotheses  ‒ discussion of the implications of exploratory analyses in terms of both substantive findings and error rates that may be uncontrolled | Para 556-565 |
| 7.2 Similarity of Results | Discuss similarities and differences between reported results and work of others | Para 569-600 |
| 7.3 Interpretation | Provide an interpretation of the results, taking into account‒ sources of potential bias and threats to internal and statistical validity‒ imprecision of measurement protocols‒ overall number of tests or overlap among tests‒ adequacy of sample sizes and sampling validity | Para 602-613 |
| 7.4 Generalizability | Discuss generalizability (external validity) of the findings, taking into account  ‒ target population (sampling validity)  ‒ other contextual issues (setting, measurement, time; ecological validity | Para615-622 |
| 7.5 Implications | Discuss implications for future research, program, or policy | 623-636 |
